# Theranostics in Radiation Medicine: Integrating Radiopharmaceutical Therapy and External-Beam Radiotherapy

**DOI:** 10.3390/jcm15166339

**Published:** 2026-08-17

**Authors:** Senthamizhchelvan Srinivasan, Joseph A. Moore, Sarah Han-Oh

**Affiliations:** 1 Department of Radiation Medicine, School of Medicine, Indiana University, Indianapolis, IN 46202, USA; sensrini@iu.edu; 2 Department of Radiation Oncology, School of Medicine, University of Minnesota, Minneapolis, MN 55455, USA; jamoore@umn.edu; 3 Department of Radiation Oncology, School of Medicine, Johns Hopkins University, Baltimore, MD 21287, USA

**Keywords:** theranostics, radiopharmaceutical therapy, external-beam radiotherapy, molecular imaging, dosimetry, biologically effective dose

## Abstract

Theranostics has transformed nuclear medicine from an imaging-focused discipline into a data-rich radiation medicine platform in which target expression, pharmacokinetics, tumor dose, and response can be measured in the same patient. This critical narrative review evaluates patient-specific integration of radiopharmaceutical therapy (RPT) with external-beam radiotherapy (EBRT), with emphasis on quantitative imaging, absorbed-dose estimation, spatial registration, biological interpretation, adaptation thresholds, and reporting. We performed a targeted search of PubMed/MEDLINE, ClinicalTrials.gov, U.S. Food and Drug Administration records, professional-society guidance, and reference lists for English-language evidence available through 31 July 2026, prioritizing guidelines, regulatory documents, randomized and prospective trials, technical validation studies, and clinically informative retrospective series. Established RPT platforms are distinguished from investigational combined-modality applications and emerging or speculative technologies. Liver-directed Y-90 radioembolization combined with focal EBRT remains the most developed model, whereas head and neck, prostate, meningioma, lymphoma, and bone-dominant strategies illustrate distinct clinical geometries and levels of readiness. Across platforms, direct addition of absorbed dose in gray (Gy) is a geometric description, not automatically a biological endpoint; biologically effective dose (BED) and equivalent dose in 2-Gy fractions (EQD2) should be treated as model-based estimates with explicit assumptions. Future theranostic radiation medicine should therefore be built on prospective trials with prespecified dosimetry, uncertainty analysis, adaptation rules, and shared cross-modality reporting standards.

## 1. Introduction

Theranostics links diagnosis and therapy through a shared molecular target, a matched or closely related radiopharmaceutical, and an imaging signal that can guide treatment decisions. In oncology, the concept has become clinically evident through somatostatin receptor (SSTR)-directed peptide receptor radionuclide therapy (PRRT) for neuroendocrine tumors (NETs), prostate-specific membrane antigen (PSMA)-directed radioligand therapy for prostate cancer, radioiodine for differentiated thyroid cancer, iobenguane for pheochromocytoma/paraganglioma (PPGL), and bone-seeking or liver-directed radionuclide therapies. The unique advantage is not simply that radioactive drugs treat cancer, but that biological targeting and serial imaging allow the radiation dose distribution to be at least partly measurable in each patient [1,2,3,4,5,6]. This is important for radiation medicine because RPT is increasingly being used before, after, or during EBRT. These two distinct modalities have different but complementary strengths. EBRT provides geometrically localized treatment to the tumor with high conformity, while RPT offers systemic therapy through a biologically distributed intervention that can reach multifocal disease, microscopic disease, or anatomically difficult targets when sufficient target uptake exists. The combined strategy is therefore attractive when the modalities solve different parts of the same clinical problem, such as diffuse intrahepatic tumor plus a dominant lesion, recurrent head and neck cancer near dose-limited structures, pelvic or metastasis-directed treatment in prostate cancer, or SSTR-positive meningioma adjacent to critical neural structures [7,8,9,10,11,12].

The main challenge is that RPT and EBRT cannot be combined reasonably by absorbed dose alone. RPT absorbed dose is governed by biodistribution, residence time, target density, clearance, particle range, and microdistribution. EBRT dose is governed by beam arrangement, fractionation, image guidance, setup uncertainty, and planned target geometry. Combination planning must therefore distinguish three related but distinct questions: (1) where energy was deposited; (2) what biological effect it is expected to produce; and (3) how, if at all, the EBRT plan should be adapted during the treatment course [5,6,7,8,9,10,11,12].

This critical narrative review focuses on patient-specific integration of RPT and EBRT rather than providing a comprehensive catalog of theranostic agents. Its central purpose is to offer a radiation-oncology framework for selecting clinically meaningful combined-modality scenarios, quantifying and registering RPT dose, interpreting biological effect, deciding when EBRT adaptation is descriptive or prescriptive, and reporting uncertainty. Included evidence had to address at least one of the following: patient selection, quantitative imaging, RPT absorbed dose, biological dose conversion, EBRT adaptation, organ-at-risk (OAR) assessment, toxicity, or multidisciplinary implementation. Throughout this review, RPT is the preferred term for therapeutic radiopharmaceutical delivery, while molecular radiotherapy, radionuclide therapy, targeted radionuclide therapy, PRRT, radioembolization, and radioligand therapy remain common in disease-specific communities. The term theranostics is used broadly to denote a paired diagnostic-therapeutic strategy based on a shared target or pharmacologic behavior, not necessarily an identical chemical structure.

### Review Methods and Evidence Framework

Literature identification was completed through a targeted search of PubMed/MEDLINE, ClinicalTrials.gov, U.S. Food and Drug Administration regulatory records, professional-society guideline repositories, and reference lists from included articles. English-language records from database inception through 31 July 2026 were considered. Search concepts combined theranostics, radiopharmaceutical therapy, molecular radiotherapy, radioligand therapy, PRRT, radioembolization, EBRT, stereotactic body radiotherapy (SBRT), dosimetry, BED, EQD2, image registration, and agent- and disease-specific terms. Regulatory documents, society guidelines, randomized and prospective trials, technical dosimetry or validation studies, and clinically informative retrospective series were prioritized. Purely diagnostic reports without relevance to treatment selection, dosimetry, planning, response, or toxicity; non-English reports lacking sufficient methodological detail; and preclinical work when clinical data were available were not emphasized. Time-sensitive regulatory and trial-status statements were verified against primary records available on 31 July 2026.

This was a critical narrative review rather than a systematic review; no meta-analysis or formal risk-of-bias score was performed. Evidence was interpreted using four pragmatic maturity categories: (1) established clinical RPT platform; (2) established RPT with investigational RPT–EBRT integration; (3) early clinical or hypothesis-generating evidence; and (4) preclinical or speculative application. These categories reflect readiness for combined-modality decision-making, not a formal grading system. Within each category, priority was given to evidence that linked imaging or dosimetry to a prespecified treatment action, toxicity, or patient outcome rather than to contour change alone.

## 2. Molecular and Radiopharmaceutical Foundations

Most theranostic radiopharmaceuticals combine three functional components: a targeting vector, a radionuclide, and a linker or chelator that determines labeling stability and in vivo behavior. The targeting vector may be a small molecule, peptide, antibody, antibody fragment, transporter substrate, nanoparticle, or microsphere. The radionuclide determines photon or positron-imaging properties, emission type, half-life, particle range, and dosimetric feasibility. Clinical performance depends on the integrated pharmacology of the entire construct, not on target expression alone [1,2,3,4].

Diagnostic radionuclides are selected for image quality, logistics, and quantitative reliability; therapeutic radionuclides are selected for dose delivery and radiobiological effect. Beta emitters such as Lu-177, Y-90, and I-131 provide crossfire over millimeter-scale distances and can compensate partly for heterogeneous uptake. Alpha emitters such as Ra-223 and Ac-225 deliver high-linear-energy-transfer (LET) radiation over short ranges of approximately 40–100 ms (about 2–10 cell diameters) and may be advantageous for micrometastatic or cell-cluster disease when uptake is adequate. Auger and conversion electrons, including those from Tb-161, have very short path lengths (approximately 0.5–30 ms) and may provide cell-level intensification when intracellular localization is favorable [1,2,3,4,13,14,15,16,17,18,19,20].

A positive theranostic scan should not be reduced to a binary interpretation. Uptake intensity, lesion-to-background ratio, intrapatient heterogeneity, target-negative discordant lesions, tumor volume, renal or salivary uptake, marrow reserve, previous systemic therapy, and prior EBRT all influence the therapeutic index. The modern theranostic question is therefore whether enough tumor dose can be delivered safely in this patient and what additional local radiation, if any, remains necessary [5,6,21,22,23,24,25,26,27,28,29,30,31].

## 3. Established Clinical Platforms

The platforms in this section are not equivalent in evidentiary maturity. SSTR-directed PRRT, PSMA radioligand therapy, radioiodine, iobenguane, and Ra-223 have established disease-specific clinical roles; however, evidence that patient-specific RPT dose should alter an EBRT prescription is much less mature. Established efficacy of an RPT platform should therefore not be conflated with readiness for prescriptive RPT–EBRT dose accumulation.

### 3.1. SSTR-Directed PRRT

SSTR-directed PRRT is a leading proof of principle for therapeutic theranostics. In the NETTER-1 trial, patients with progressive metastatic midgut neuroendocrine tumors treated with Lu-177-DOTATATE plus standard-dose octreotide had an estimated 20-month progression-free survival of 65.2%, compared with 10.8% with high-dose octreotide, and an objective response rate of 18% versus 3% [21]. Final NETTER-1 follow-up showed numerically longer median overall survival with Lu-177-DOTATATE, although the comparison was affected by crossover and subsequent therapies [22]. Regulatory approval of Lu-177-DOTATATE made SSTR imaging a practical gate to systemic radiopharmaceutical therapy [23].

The SSTR platform is also instructive for combination planning. Patients may have widespread receptor-positive disease treated systemically, while a dominant lesion, threatened spinal canal, painful bone metastasis, or organ-compromising mass may still require EBRT. Conversely, patients who have had previous EBRT may need PRRT dosimetry to avoid excessive marrow, kidney, or other OAR exposure. For these reasons, PRRT should be understood as part of a radiation continuum rather than as an isolated nuclear medicine administration; nevertheless, evidence for prescriptive PRRT–EBRT dose integration remains limited [5,6,21,22,23].

### 3.2. PSMA-Directed Radioligand Therapy

PSMA-directed theranostics has transformed advanced prostate cancer management. In the VISION trial, patients with PSMA-positive metastatic castration-resistant prostate cancer (mCRPC) previously treated with an androgen receptor pathway inhibitor and taxane chemotherapy had improved median overall survival with Lu-177-PSMA-617 plus standard care versus standard care alone (15.3 versus 11.3 months) [24]. PSMAfore moved the platform earlier, demonstrating improved radiographic progression-free survival compared with a change in androgen receptor pathway inhibitor therapy in taxane-naïve patients after one prior androgen receptor pathway inhibitor [25]. On 28 March 2025, the U.S. Food and Drug Administration expanded the indication for lutetium Lu-177 vipivotide tetraxetan to adults with PSMA-positive mCRPC previously treated with an androgen receptor pathway inhibitor and considered appropriate to delay taxane-based chemotherapy [26]. Health-related quality-of-life, pain, and symptomatic skeletal-event analyses support the clinical relevance of this earlier-line positioning [27].

On 31 July 2026, the U.S. Food and Drug Administration further approved lutetium Lu 177 vipivotide tetraxetan in combination with androgen receptor pathway inhibitor therapy for adults with PSMA-positive metastatic androgen pathway modulation-naïve or -sensitive (mAPMN/S) prostate cancer (previously referred to as metastatic hormone-sensitive prostate cancer), with patient selection based on an approved PSMA PET product. In the randomized, multicenter, open-label PSMAddition trial (NCT04720157; *n* = 1144), patients received lutetium Lu 177 vipivotide tetraxetan 7.4 GBq (200 mCi) every 6 weeks for six doses plus androgen receptor pathway inhibitor therapy or androgen receptor pathway inhibitor therapy alone. The combination significantly improved radiographic progression-free survival by blinded independent central review (hazard ratio, 0.72; 95% confidence interval, 0.58–0.90; *p* = 0.002); median radiographic progression-free survival was not reached in either arm, overall survival data were immature, and adverse reactions were consistent with prior experience [32].

For radiation medicine, PSMA is especially important because imaging can be used at several decision points: staging, EBRT target delineation, selection for RPT, assessment of target-negative disease, response evaluation, and planning of metastasis-directed EBRT. The same patient may receive pelvic EBRT, prostate-bed salvage EBRT, stereotactic treatment to oligometastases, and systemic PSMA RPT over the disease course. Cumulative OAR doses to marrow, salivary glands, kidneys, and other previously irradiated tissues, together with lesion-level dose, therefore become clinically relevant as RPT moves into earlier lines, including mAPMN/S disease, with longer expected survival. Current clinical data, however, do not establish a validated dose-subtraction rule for EBRT [24,25,26,27,32,33,34,35].

### 3.3. Radioiodine and Transporter-Based Theranostics

Radioiodine remains the prototype clinical theranostics. Diagnostic iodine imaging, thyroglobulin trends, post-therapy scans, and clinical risk factors guide differentiated thyroid cancer treatment across remnant ablation, adjuvant therapy, surveillance, and metastatic disease management. Modern innovation has not displaced this paradigm; rather, it has renewed it through redifferentiation strategies intended to restore iodine uptake in selected radioiodine-refractory tumors [28,29].

Transporter-based theranostics also includes high-specific-activity I-131-iobenguane for metaiodobenzylguanidine (MIBG)-avid unresectable or metastatic PPGL. U.S. Food and Drug Administration approval was supported by a phase 2 experience in which durable reduction in antihypertensive medication burden and radiographic responses occurred in a subset of patients [30,31]. From a radiation-planning perspective, these therapies illustrate a general principle: a biological uptake mechanism can define the therapeutic field more accurately than anatomy alone, but the delivered dose remains patient-specific and must be interpreted alongside marrow and normal-organ tolerance.

### 3.4. Bone-Targeted and Alpha-Emitting Therapy

Ra-223 dichloride established the survival relevance of an alpha-emitting bone-targeted therapy in metastatic prostate cancer with symptomatic bone metastases [13]. Alpha emitters have since expanded into targeted constructs such as Ac-225-PSMA ligands and antibodies, with early clinical studies and multicenter retrospective data showing activity in heavily pretreated mCRPC while also highlighting toxicity challenges such as xerostomia and marrow suppression [14,15,16]. Outside the established disease-specific use of Ra-223, most targeted alpha constructs remain investigational.

The appeal of alpha therapy in combined-modality radiation medicine is its high-LET, short-range energy deposition. This may complement EBRT in microscopic or marrow-sparing settings, but it also makes microdistribution decisive. Unlike photon EBRT, a uniform organ mean dose may poorly represent cellular hit probability for short-range particles. The field therefore needs not only organ-level absorbed-dose reporting, but also radiobiologic models that can relate uptake pattern, particle range, repair, and toxicity to clinical decisions [13,14,15,16,36,37,38,39,40,41,42,43,44,45].

## 4. Emerging Theranostic Platforms

Fibroblast activation protein (FAP)-targeted ligands have attracted substantial interest because FAP is expressed in cancer-associated fibroblasts across many solid tumors. Early FAP inhibitor (FAPI)-targeted RPT experiences, including Lu-177-EB-FAPI in metastatic radioiodine-refractory thyroid cancer and first-in-human FAPI-XT studies, suggest feasibility while underscoring the need for durable tumor retention and normal-tissue safety data [17,18]. These agents are especially relevant to EBRT integration because many FAP-avid tumors are anatomically complex, locally advanced, or previously irradiated; however, the current evidence is early clinical and hypothesis-generating.

Terbium radionuclides provide a compelling theranostic concept because different isotopes can support PET, SPECT, beta, alpha, and Auger-like therapeutic applications. Tb-161 is particularly interesting as a beta and conversion/Auger electron emitter; early PSMA-targeted clinical data suggest feasibility and motivate further dose-effect work [19,20]. Nanoparticle and supramolecular radiopharmaceuticals add another layer of design control by altering circulation, multivalency, clearance, and payload delivery [46,47]. Their value for combined RPT–EBRT care will depend on whether these engineering advantages translate into predictable tumor dose, controllable normal-organ exposure, and feasible quantitative imaging. At present, these approaches are not ready for routine prescriptive EBRT adaptation.

Table 1 summarizes representative theranostic pairs, evidence maturity, and radiation-medicine integration issues.

## 5. Dosimetry: From Administered Activity to Absorbed Dose

Administered activity is not equal to absorbed dose. Patient-specific RPT dosimetry requires quantitative imaging, system calibration, corrections for recovery and physical effects, time–activity modeling, absorbed-dose calculation, and uncertainty evaluation. Quantitative single-photon emission computed tomography/computed tomography (SPECT/CT) and positron emission tomography/computed tomography (PET/CT) are essential because most clinically relevant therapeutic distributions are heterogeneous across organs, lesions, and substructures [5,6,46,47,48,49,50,51,52,53].

Using standard Medical Internal Radiation Dose (MIRD) terminology, activity at time t, time-integrated activity, absorbed-dose rate, and absorbed dose are distinct quantities. For a target region $r_{T}$ receiving energy from source regions $r_{S}$, the relationships are: 

Instantaneous absorbed-dose rate

(1) $$\dot{D}\left(r_{T},t\right)=\sum_{r_{S}}A\left(r_{S},t\right)S\left(r_{T}\leftarrow r_{S}\right)$$

Time-integrated activity and absorbed dose

(2) $$\tilde{A}\left(r_{S}\right)=\int_{0}^{\infty }A\left(r_{S},t\right)\;dt$$

(3) $$D\left(r_{T}\right)=\int_{0}^{\infty }\dot{D}\left(r_{T},t\right)\;dt=\sum_{r_{S}}\tilde{A}\left(r_{S}\right)S\left(r_{T}\leftarrow r_{S}\right)$$

Here, *A*$\left(r_{S},t\right)$ is activity in source region $r_{S}$ at time t [Bq]; the time-integrated activity in $r_{S}$ is the time integral of *A*$\left(r_{S},t\right)$ [Bq·s or Bq·h]; $S\left(r_{T}\leftarrow r_{S}\right)$ is the mean absorbed dose to $r_{T}$ per nuclear transformation in $r_{S}$ [Gy/(Bq·s)] or equivalent units; the overdot on D denotes absorbed-dose rate [Gy/s]; and D is absorbed dose [Gy]. The standard S-value summation assumes a defined geometry and mass. When anatomy, mass, or activity distribution changes over time, time-dependent S values or direct voxel/Monte Carlo integration may be required. These expressions describe mean or voxel-absorbed dose and do not, by themselves, account for biological effect or subcellular microdistribution [50,51,52,53].

The practical burden in obtaining absorbed dose in RPT is nontrivial. Multi-time-point imaging provides more robust time-integrated activity than single-time-point methods, but it increases patient inconvenience, camera use, staffing demands, and costs. Recent work has therefore focused on workflow simplification, including optimized sparse imaging schedules, two-time-point methods, population pharmacokinetic approaches, and artificial intelligence (AI)-assisted segmentation or prediction [46,47,48,49,54,55,56]. These methods should not be treated as interchangeable: each has assumptions that must be validated for the isotope, agent, tumor type, scanner, reconstruction protocol, and endpoint of interest, and any bias introduced by simplification should be reported.

For EBRT integration, the dose map must be placed into the treatment-planning geometry. This step includes rigid and, where appropriate, deformable registration of post-therapy SPECT/PET/CT to the EBRT planning CT or magnetic resonance imaging (MRI), contour harmonization, dose-grid resampling, and quality assurance (QA) by a physicist familiar with both modalities. Deformable registration should not be treated as ground truth; QA must be region- and decision-specific [57]. The most important planning question should drive the level of sophistication. If the question is whether liver mean dose remains within a conservative normal-tissue limit, structure-level dosimetry may suffice. If the question is whether RPT dose replaces EBRT fractions near the spinal cord, optic pathway, salivary gland, brainstem, or mucosa, voxel-level methods and more explicit uncertainty analysis are needed [8,9,10,11,12,57,58,59,60,61,62].

Total uncertainty should be propagated from calibration, acquisition, reconstruction, segmentation, time–activity fitting, mass estimation, the dose engine, registration, and any biological conversion. A point estimate without an uncertainty statement is insufficient for prescriptive adaptation [53,57].

## 6. Radiobiology of EBRT and RPT

EBRT and RPT share the same absorbed dose, but they do not share the same temporal, spatial, or microscopic exposure pattern. EBRT usually delivers high-dose-rate fractions over minutes, separated by hours or days for repair and normal-tissue recovery. RPT often delivers continuously over hours to days as radionuclides decay and biologic clearance changes local activity. The total dose-rate history may vary across lesions and organs in the same patient [8,9,10,11,12,36,37,38,39,40,41].

The linear-quadratic (LQ) model remains the most common model-based framework for translating dose and fractionation into biological effect. For conventional low-LET photon EBRT, a simplified expression is:

(4) $${BED}_{EBRT}=nd\left(1+\frac{d}{\alpha /\beta }\right)$$

where n is the number of fractions, d is dose per fraction, and alpha/beta reflects tissue- or tumor-specific fractionation sensitivity. For protracted low-LET RPT, a simplified expression is:

(5) $${BED}_{RPT}=D\left(1+\frac{GD}{\alpha /\beta }\right)$$

where D is absorbed dose and G is the Lea–Catcheside repair-weighting factor, which depends on the complete dose-rate history and repair half-time. One expression for G and the corresponding model-derived EQD2 are:

(6) $$G=\frac{2}{D^{2}}\int_{0}^{T}\dot{D}\left(t\right)\left[\int_{0}^{t}\dot{D}\left(w\right)e^{-\mu \left(t-w\right)}\;dw\right]dt,\;\;$$

(7) $$EQD2=\frac{BED}{1+2/\left(\alpha /\beta \right)}$$

(8) EQD2RPT=DRPTαβ+G∞·DRPTαβ+2

(9) EQD2EBRT=DEBRT·αβ+dEBRTαβ+2αβ+dEBRTαβ+2

where μ is the repair rate, assuming that the probability of a repair event decreases exponentially as a function of time; w is the time of the first single-strand DNA break; t is the time of the second break; and T is the duration of irradiation. Equieffective dose (EQD2 in Gy) is a quantity that, like BED, is intended to account for differences in fractionation or dose rate; EQD2 refers to a reference value of 2 Gy per fraction. BED is equivalent to EQD0 and thus particularly relevant to RPT. Low, continuous dose rates delivered by radiopharmaceuticals require modification of this equation to incorporate Lea–Catcheside time factor G (T = ∞). These expressions are bookkeeping models, not proof of biological equivalence. BED or EQD2 values should be summed only when the compared doses refer to the same tissue endpoint, spatial scale, alpha/beta, repair model, and radiation quality assumption. When these conditions are not met, physical dose maps and model outputs should be reported separately [8,9,10,11,12,36,37,38,39,40,41,42,43,44,45].

Several limitations are especially important. First, alpha/beta values are often extrapolated from photon fractionation data and may not capture RPT-specific biology. Second, the repair half-time and complete dose-rate history are rarely known with precision. Third, tumor and normal tissue receive nonuniform doses at voxel and subvoxel scales. Fourth, relative biological effectiveness (RBE), LET, particle range, cellular targeting, and subcellular microdistribution may dominate for alpha and Auger/conversion electron emitters, making a photon-derived LQ conversion or a single-organ mean dose misleading. Fifth, the same organ mean dose may have different implications depending on which substructures or stem-cell compartments are irradiated. EQD2 should therefore be labeled as model-derived and should not be used as a validated equivalence or routine fraction-subtraction rule unless supported by agent-, tissue-, and endpoint-specific clinical data [36,37,38,39,40,41,42,43,44,45].

Table 2 summarizes key differences between EBRT and RPT that affect cumulative dose assessment.

## 7. Practical Framework for RPT–EBRT Integration

A combined RPT–EBRT plan should start with a clinical hypothesis. Examples include reducing EBRT dose to a previously irradiated mucosal surface because tumor-selective RPT has delivered meaningful dose, escalating SBRT to a residual cold region after Y-90 radioembolization, using PSMA RPT to cover microscopic disease while EBRT treats macroscopic oligometastases, or using PRRT before fractionated EBRT to intensify dose to SSTR-positive meningioma. Without a hypothesis, dose accumulation risks becoming a mathematical exercise that does not change patient care [7,8,9,10,11,12,33,34,35,58,59,60,61,62,63,64,65,66,67,68,69].

Combined RPT–EBRT dosimetry comprises at least four distinct scenarios: (1) regional overlapping dose maps, exemplified by Y-90 liver therapy followed by focal EBRT; (2) same-target overlap, such as iopofosine or PRRT combined with EBRT, where target and OAR doses may overlap spatially; (3) systemic–focal spatial cooperation, such as PSMA RPT or bone-targeted therapy plus site-directed SBRT, where voxelwise dose subtraction is often not the main question; and (4) cumulative OAR assessment across prior and current radiation courses. These scenarios differ in imaging reliability, temporal separation, registration validity, OAR relevance, and clinical intent and should not be analyzed as a single dosimetric problem.

The safest workflow includes: (1) define the clinical decision and relevant target/OAR endpoints; (2) classify the scenario; (3) acquire quantitative pre-therapy and post-therapy imaging using a calibrated protocol; (4) calculate RPT absorbed dose at the granularity required by the decision; (5) register dose maps to the EBRT planning data set and review registration and contour uncertainty; (6) choose physical dose, BED/EQD2, or no biological conversion according to the decision and available validation; (7) classify confidence as low, intermediate, or high; (8) select no adaptation, descriptive adaptation, or prescriptive adaptation; and (9) document uncertainty and follow-up toxicity. This workflow should be implemented by a multidisciplinary team rather than delegated to either nuclear medicine or radiation oncology alone [5,6,7,8,9,10,11,12,33,34,35,46,47,48,49,50,51,52,53,57,58,59,60,61,62,70,71].

**Figure 1:**
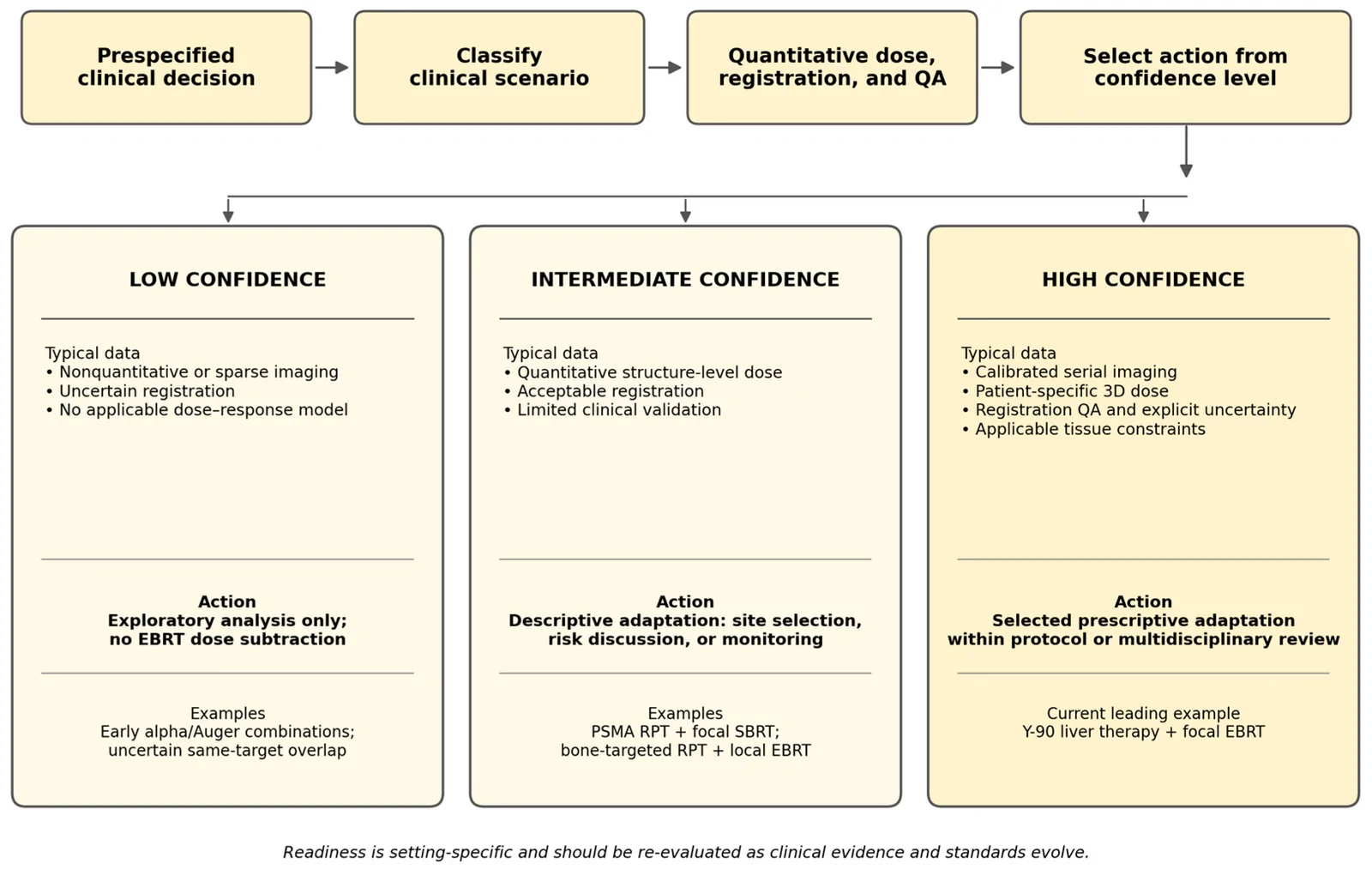
Confidence-based framework for using RPT dose in EBRT planning.

In practice, the clinician often faces three possible outputs as shown in Figure 1. Low-confidence use is exploratory: RPT is delivered, but EBRT proceeds as planned because dose contribution, registration, or dose–response validity is too uncertain. Intermediate-confidence use is descriptive: RPT dose informs risk discussion, toxicity monitoring, or selection of sites for focal EBRT without formal biological subtraction. High-confidence use may be prescriptive: RPT dose directly changes EBRT dose, fraction number, target coverage, or OAR constraints. Prescriptive adaptation requires calibrated serial imaging, patient-specific three-dimensional dose, registration QA, explicit uncertainty, an applicable clinical dose–response framework, and prospective protocol or multidisciplinary oversight. The examples indicated in Figure 1 are current relative readiness and are not universal recommendations.

## 8. Disease-Specific Synthesis

### 8.1. Liver-Directed Y-90 and Focal EBRT

The liver is the strongest current template for planned RPT–EBRT dosimetry because Y-90 microspheres create a measurable, heterogeneous intrahepatic dose distribution that can be imaged after treatment and integrated with focal EBRT planning. Wang and colleagues described combined Y-90 selective internal radiation therapy (SIRT) and EBRT in hepatocellular carcinoma from clinical and dosimetric perspectives, and subsequent inverse-planning work showed how SBRT could be shaped after radioembolization to account for delivered tumor and normal liver dose [58,59]. Dose–response modeling studies further illustrate why normal liver and tumor control assumptions can materially change planning conclusions [60]. This evidence is technically informative but does not constitute randomized proof that composite-dose adaptation improves clinical outcomes.

The liver paradigm is important beyond hepatocellular carcinoma. It demonstrates that RPT is not always systemic drug therapy; it can be a regional radiation intervention with a post-therapy dose map. When a residual lesion remains, EBRT can be used to boost underdosed areas. When normal liver has already received substantial microsphere dose, EBRT may need to be de-escalated or reshaped. This is the most concrete example of RPT informing an EBRT plan rather than simply being counted as previous treatment. Selected regional overlapping-dose cases may support prescriptive adaptation, but prospective outcome data and validated composite constraints remain limited.

### 8.2. Head and Neck Re-Irradiation

Recurrent head and neck cancer is a compelling but high-risk setting because EBRT re-irradiation is constrained by mucosa, salivary glands, spinal cord, mandible, swallowing structures, carotid arteries, optic pathways, and prior dose. Adam and colleagues developed a voxel-level dosimetry framework for I-131 radiopharmaceutical therapy combined with EBRT, showing how Monte Carlo RPT dose maps could be brought into EBRT planning paradigms [61]. A phase 1 study of I-131 iopofosine with EBRT in recurrent or metastatic head and neck cancer provided early clinical safety and activity data [62].

This setting illustrates the highest-stakes version of combined dosimetry: the question is not only how much total dose was delivered, but whether RPT dose in tumor-adjacent normal tissue should modify re-irradiation. Because serial anatomy, prior dose, tumor shrinkage, and organ motion can all be uncertain, current evidence supports exploratory or descriptive use. Routine prescriptive fraction replacement is not established and should be approached cautiously with detailed uncertainty analysis.

### 8.3. Prostate Cancer

Prostate cancer is likely to become a common RPT–EBRT integration setting because many patients receive EBRT at several phases of disease and PSMA radioligand therapy has moved earlier, including the 31 July 2026 FDA approval of lutetium Lu-177 vipivotide tetraxetan with androgen receptor pathway inhibitor therapy for PSMA-positive mAPMN/S disease [32]. PROQURE-I addressed concurrent Lu-177-PSMA-617, EBRT, and androgen deprivation therapy (ADT) in node-positive prostate cancer, while lesion-level dosimetry studies have explored Lu-177-PSMA-617 with SBRT in oligometastatic castration-sensitive disease [33,34]. Contemporary reviews frame the combination of EBRT and Lu-177-labeled PSMA ligands as a developing field with specific trial, dosimetry, and toxicity questions [35].

Several use cases are plausible. PSMA RPT may treat occult systemic disease while SBRT ablates visible oligometastases. Pelvic EBRT may sterilize nodal basins while RPT treats PSMA-positive disease outside the field. RPT dose maps may identify lesions that have already received meaningful dose and lesions that remain cold or undertreated. In current practice, this is generally systemic–focal spatial cooperation and site selection rather than validated voxelwise subtraction. Available evidence does not yet establish that PSMA RPT dose-guided EBRT adaptation improves outcomes. Marrow exposure, salivary toxicity, renal uptake, fracture risk, and cumulative EBRT history all shape patient selection.

### 8.4. Meningioma and Other SSTR-Positive Tumors

Advanced meningioma provides a biologically rational example because SSTR expression can be imaged and targeted, while EBRT remains a central local therapy. Kreissl and colleagues reported PRRT combined with fractionated EBRT in advanced symptomatic meningioma, and long-term follow-up of the same multimodal strategy suggested feasibility and durable stabilization in a small cohort [63,64].

The dosimetric opportunity in meningioma differs from systemic metastatic disease. Many tumors are near optic nerves, chiasm, cranial nerves, brainstem, pituitary, cochlea, or normal brain. Even a modest RPT contribution may matter if it overlaps a high-risk substructure or if it supports EBRT dose de-escalation in a previously treated region. However, the evidence base remains small and largely nonrandomized, and no validated absorbed-dose or biological threshold supports routine EBRT dose subtraction. PRRT–EBRT planning should therefore be individualized, transparent, and preferably protocol-based.

### 8.5. Lymphoma, Bone Metastases, and Historical Combined-Modality Lessons

Historical lymphoma experience with EBRT followed by Y-90 ibritumomab tiuxetan demonstrated spatial cooperation: focal EBRT could address bulky disease that was likely to respond less well to systemic radioimmunotherapy, while RPT treated disseminated disease [65]. Bone-metastasis studies with Sm-153 plus local EBRT and trials of Ra-223 plus stereotactic ablative radiotherapy (SABR) similarly emphasize the complementary logic of systemic skeletal irradiation and focal high-dose treatment [66,67,68]. A metastatic osteosarcoma experience with Ra-223, systemic agents, and EBRT further illustrates clinical feasibility in a rare, high-need setting, although evidence remains limited [69].

These reports are important because they broaden the discussion beyond modern PSMA and PRRT. They show that the clinical intuition behind combination therapy has existed for decades. Their principal lesson is spatial cooperation and sequencing rather than validated voxel-level cumulative dosimetry. Modern molecular imaging, quantitative reconstruction, and computational planning now create an opportunity to measure, adapt, and report these combinations more rigorously.

Table 3 classifies selected disease settings by dosimetric scenario and current readiness.

## 9. Artificial Intelligence, Automation, and Infrastructure

Theranostic radiation medicine is data-intensive. Quantitative imaging, segmentation, time–activity fitting, Monte Carlo or dose-kernel calculations, deformable registration, plan adaptation, and toxicity modeling all create opportunities for automation. The most clinically mature AI uses are workflow assistance, such as organ or lesion segmentation and QA triage, whereas sparse-time-point kinetic or dose prediction, deformable dose accumulation, radiomics-based outcome prediction, and automated EBRT plan adaptation remain largely investigational [54,55,56].

However, automation should not weaken accountability. Models used for RPT dosimetry or EBRT adaptation must be auditable, validated on appropriate scanner and reconstruction protocols, externally tested when possible, and evaluated against clinically meaningful endpoints. An algorithm that performs well for organ segmentation may fail for small lesions; a dose predictor trained on fixed-activity therapy may not generalize to adaptive dosing; and a deformable registration algorithm may produce visually plausible but dosimetrically misleading maps near surgical defects or tumor shrinkage. Human review by trained nuclear medicine physicians, radiation oncologists, and medical physicists remains essential.

Institutional infrastructure is as important as computation. A credible RPT–EBRT program needs radiopharmacy support, calibrated quantitative imaging, therapy rooms and radiation-safety procedures, a dosimetry workflow, treatment-planning system compatibility, multidisciplinary contour review, prospective toxicity capture, and a shared language for communicating uncertainty. In many centers, the limiting step will not be a new radiopharmaceutical, but the absence of a practical bridge between nuclear medicine dosimetry and EBRT planning systems.

## 10. Reporting Standards and Research Objectives

The literature would be more interpretable if combined RPT–EBRT studies reported a minimum data set. The checklist in Table 4 is not a substitute for existing standards; it links MIRD nomenclature and quantitative RPT recommendations [50,51,52,53] with AAPM Task Group 132 registration QA [57] and International Commission on Radiation Units and Measurements (ICRU) EBRT and stereotactic-treatment reporting frameworks [70,71]. Reports should include the clinical sequence, all prior radiation, target and OAR definitions, RPT agent and administered activity, imaging time points, scanner calibration, reconstruction settings, activity quantification method, time–activity curve model, absorbed-dose calculation method, dose-grid resolution, registration method, biological conversion assumptions, uncertainty, adaptation rules, toxicity grading, and follow-up duration. Trial reports should prespecify whether dosimetry is exploratory, descriptive, or prescriptive.

Dose constraints require special caution. Extrapolating EBRT constraints directly to RPT may be misleading because RPT is protracted, heterogeneous, and agent-specific. Conversely, dismissing EBRT-derived knowledge entirely would ignore decades of organ tolerance data. The most defensible approach is contextual: use EBRT constraints as reference points when appropriate, but state when RPT-specific dose-rate, substructure, microdosimetry, or clinical dose–response data are inadequate [44,45].

Prospective trials should move beyond feasibility and ask testable questions: Does RPT dose-guided EBRT adaptation improve tumor control or reduce toxicity compared with standard sequencing? Can post-therapy imaging identify lesions that need SBRT boost? Can marrow, salivary, kidney, liver, or bowel toxicity be predicted better by composite biologic dose than by administered activity? Can simplified dosimetry protocols preserve clinically relevant accuracy? Can AI reduce workload without introducing unacceptable uncertainty? These questions require collaboration among nuclear medicine, radiation oncology, radiology, medical physics, radiobiology, radiopharmacy, biostatistics, and data science.

Table 4 provides a suggested minimum reporting checklist for combined RPT–EBRT studies.

## 11. Conclusions

Theranostics is reshaping radiation medicine by making biologically targeted radiation visible, measurable, and potentially adaptable. The next step is not simply more radiopharmaceuticals or more combinations, but more rigorous integration. RPT and EBRT should be planned together when they address complementary spatial or biological problems, and cumulative dose should be interpreted with respect for differences in dose rate, heterogeneity, particle quality, repair, and normal-tissue exposure. The most mature technical model is selected Y-90 liver therapy integrated with focal EBRT, but head and neck, prostate, meningioma, lymphoma, and bone-dominant approaches show the breadth of the field. Prospective evidence that composite RPT–EBRT dose adaptation improves tumor control or reduces toxicity remains limited for most disease settings. Until validated, most PSMA, head and neck, meningioma, lymphoma, bone, alpha/Auger, and AI-enabled applications should be described as investigational or hypothesis-generating. The publication standard should move toward patient-specific quantitative imaging, transparent dosimetry, biologically explicit modeling, uncertainty reporting, and prospective trials in which RPT dose can genuinely modify EBRT decisions. If these standards are met, theranostic radiation medicine can evolve from empiric sequencing to individualized, evidence-based multimodality radiation planning.

## Figures and Tables

**Table 1:** Representative theranostic platforms, evidence maturity, and radiation-medicine integration issues.

Platform/Target	Common Imaging-Therapy Pair	Evidence Maturity/Current Use	Radiation-Medicine Integration Issue
Sodium-iodide symporter/iodine handling	I-123, I-124, or diagnostic I-131 imaging; I-131 therapy	Established disease-specific platform; redifferentiation remains active in selected refractory disease.	Usually individualized sequencing; prior neck EBRT, airway risk, marrow reserve, and disease distribution influence local-therapy decisions.
SSTR in NET	Ga-68-DOTATATE/DOTATOC PET; Lu-177-DOTATATE PRRT	Established phase 3/approved RPT for SSTR-positive GEP-NETs; combined EBRT dose adaptation is investigational.	Serial SPECT/CT dosimetry, renal and marrow risk, and focal EBRT for dominant lesions require coordinated planning.
PSMA in prostate cancer	Ga-68-PSMA-11 or F-18 PSMA PET; Lu-177-PSMA-617; investigational Ac-225 and Tb-161 agents	Approved for selected PSMA-positive mCRPC and, from 31 July 2026, with androgen receptor pathway inhibitor therapy for PSMA-positive mAPMN/S disease; RPT–EBRT adaptation remains investigational.	PET guides eligibility and lesion selection; post-therapy dose generally informs descriptive rather than prescriptive EBRT decisions.
Norepinephrine transporter/MIBG uptake	I-123-MIBG imaging; high-specific-activity I-131-iobenguane	Approved for scan-positive unresectable or metastatic PPGL; combined dosimetry is uncommon.	Catecholamine risk, marrow dose, prior EBRT, and normal-organ exposure require multidisciplinary monitoring.
Bone mineral turnover	Bone scintigraphy or PSMA PET as appropriate; Ra-223 dichloride or historical Sr-89/Sm-153 agents	Ra-223 has an established role in selected prostate cancer; combined focal EBRT strategies have mixed evidence.	Marrow reserve, fracture risk, disease burden, and symptomatic sites determine sequencing.
Hepatic arterial tumor supply	Angiography, Tc-99m-MAA planning, Y-90 PET/SPECT or bremsstrahlung imaging; Y-90 microspheres	Mature regional platform; strongest current template for spatial RPT–EBRT integration.	Post-therapy dose maps may support selected focal EBRT adaptation after registration and uncertainty review.
Fibroblast activation protein	Ga-68 or F-18 FAPI PET; Lu-177, Ac-225, or other FAP-targeted agents in trials	Early clinical/hypothesis-generating; efficacy and retention remain under study.	Normal-tissue expression, residence time, and absent validated dose–response limit prescriptive use.
Next-generation radionuclide and carrier systems	Terbium pairs, nanoparticles, supramolecular assemblies	Early clinical or translational; not ready for routine prescriptive integration.	Requires standardized production, quantitative imaging, microdosimetry, and clinical dose-effect validation.

NET, neuroendocrine tumor; PPGL, pheochromocytoma/paraganglioma.

**Table 2:** Important differences between EBRT and RPT for cumulative dose assessment.

Feature	EBRT	RPT	Implication for Combination Planning
Spatial source	External beams shaped by planning system, immobilization, image guidance, and margins	Internal sources determined by target expression, vascular delivery, clearance, and excretion	Registration and contour harmonization are essential before dose accumulation.
Temporal pattern	High dose-rate fractions, usually minutes per fraction	Protracted exposure over hours to days with changing activity concentration	Biological conversion may be explored when tissue effect drives the decision, but assumptions and validation must be explicit.
Heterogeneity	Planned heterogeneity is usually controlled and visible in the dose-volume histogram	Lesion, organ, voxel, and subcellular heterogeneity may be substantial	Organ mean dose can hide hot substructures or cold tumor regions.
Particle quality	Mostly low-LET photons or protons in routine practice	Beta, alpha, Auger/conversion electrons, or microsphere distributions depending on agent	LET, RBE, particle range, and microdistribution may preclude a universal conversion.
OAR limitation	Anatomic proximity and beam path dominate	Uptake, clearance, and whole-body distribution dominate	Toxicity patterns may be complementary but not independent.
Verification	Image guidance verifies position; in vivo dosimetry used selectively	Post-therapy imaging can verify biodistribution and support absorbed-dose estimation	RPT imaging creates an opportunity for adaptive EBRT planning.

OAR, organ at risk.

**Table 3:** Selected clinical scenarios for RPT–EBRT integration.

Disease Setting	Representative Evidence	Scenario Class and Current Readiness	Key Lesson for Dosimetry
Liver tumors treated with Y-90 SIRT plus EBRT or SBRT	Clinical dosimetry studies and inverse planning after SIRT [58,59,60]	Regional overlapping dose maps; most mature template, with selected prescriptive potential.	Post-SIRT maps may identify underdosed tumor or spared liver; composite constraints and outcome validation remain limited.
Recurrent or metastatic head and neck cancer with I-131 iopofosine plus EBRT	Voxel-level dosimetry framework and phase 1 clinical safety data [61,62]	Same-target overlap; technically advanced but exploratory/descriptive.	Dose maps may identify high-risk structures, but routine fraction replacement is not established.
Prostate cancer with PSMA RPT plus pelvic, prostate-bed, or metastasis-directed EBRT	PROQURE-I, lesion dosimetry with SBRT, and emerging reviews/trials [33,34,35]	Systemic–focal spatial cooperation; descriptive/investigational.	PET and post-therapy imaging support eligibility, site selection, and monitoring rather than validated dose subtraction.
SSTR-positive meningioma with PRRT followed by fractionated EBRT	Initial and long-term pilot cohorts [63,64]	Same-target overlap; feasible but exploratory, with small-cohort evidence.	Potential relevance near neural OARs, but no validated threshold for EBRT de-escalation.
Relapsed bulky follicular lymphoma with EBRT plus radioimmunotherapy	Historical EBRT followed by Y-90 ibritumomab tiuxetan [65]	Sequential spatial cooperation; historical descriptive evidence.	Demonstrates complementary fields; modern voxel-level cumulative dosimetry was limited.
Bone-dominant metastatic disease	Sm-153 plus local EBRT, Ra-223 plus SABR trials, and metastatic osteosarcoma experience [66,67,68,69]	Systemic skeletal plus focal EBRT; mixed descriptive/investigational evidence.	Marrow reserve, fracture risk, pain control, and subclinical disease cannot be summarized by one mean dose.

SABR, stereotactic ablative radiotherapy; SBRT, stereotactic body radiotherapy; SIRT, selective internal radiation therapy.

**Table 4:** Suggested minimum reporting checklist for combined RPT–EBRT studies, aligned with existing modality-specific standards.

Domain	Minimum Elements to Report	Why It Matters
Clinical context	Disease, stage, intent, prior systemic therapy, prior EBRT fields/doses, and treatment interval.	Defines benefit, toxicity risk, and whether summation is clinically meaningful.
Decision role	Prespecified clinical question; exploratory, descriptive, or prescriptive role; adaptation trigger.	Prevents retrospective reinterpretation and distinguishes measurement from action.
RPT administration	Agent, radionuclide, activity, cycle, supportive measures, and radiation-safety constraints.	Allows comparison across agents and cycles.
Imaging protocol	Scanner, time points, reconstruction/corrections, calibration, and recovery or partial-volume method.	Quantitative image quality is the foundation for dosimetry.
RPT dosimetry	MIRD quantities/units, segmentation, time–activity fit, mass scaling, dose engine/grid, and organ/lesion doses.	Determines whether absorbed dose is reproducible and decision-ready.
Registration and accumulation	Planning CT/MRI, rigid/deformable method, direction/region, QA, contour propagation, and resampling.	Small spatial errors can dominate conclusions near critical structures.
Radiobiology	Physical dose versus BED/EQD2; α/β, repair half-time, RBE/LET, spatial scale, and model validity.	Prevents unsupported claims of biological equivalence.
Uncertainty and QA	Calibration, segmentation, curve fit, mass, dose engine, registration, biological model, and combined uncertainty.	A point estimate alone is insufficient for prescriptive adaptation.
EBRT adaptation	Original and adapted plans; changed dose/fractionation/targets/constraints; approval process.	Makes the treatment action reproducible.
Outcomes	Local control, progression, symptoms, acute/late toxicity, patient-reported outcomes, and follow-up.	Links dosimetry to patient-centered benefit and harm.

BED, biologically effective dose; EQD2, equivalent dose in 2-Gy fractions; LET, linear energy transfer; MIRD, Medical Internal Radiation Dose; QA, quality assurance; RBE, relative biological effectiveness.

## Data Availability

No new data were created or analyzed in this study. Data sharing is not applicable to this article.

## Abbreviations

The following abbreviations are used in this manuscript:

ADT	Androgen deprivation therapy
AI	Artificial intelligence
BED	Biologically effective dose
CT	Computed tomography
EBRT	External-beam radiotherapy
EQD2	Equivalent dose in 2-Gy fractions
FAP	Fibroblast activation protein
FAPI	Fibroblast activation protein inhibitor
GEP-NET	Gastroenteropancreatic neuroendocrine tumor
LET	Linear energy transfer
LQ	Linear-quadratic
mAPMN/S	Metastatic androgen pathway modulation-naïve or -sensitive prostate cancer
mCRPC	Metastatic castration-resistant prostate cancer
MIBG	Metaiodobenzylguanidine
MIRD	Medical Internal Radiation Dose
MRI	Magnetic resonance imaging
NET	Neuroendocrine tumor
OAR	Organ at risk
PET	Positron emission tomography
PPGL	Pheochromocytoma/paraganglioma
PRRT	Peptide receptor radionuclide therapy
PSMA	Prostate-specific membrane antigen
QA	Quality assurance
RBE	Relative biological effectiveness
RPT	Radiopharmaceutical therapy
SABR	Stereotactic ablative radiotherapy
SBRT	Stereotactic body radiotherapy
SIRT	Selective internal radiation therapy
SPECT	Single-photon emission computed tomography
SSTR	Somatostatin receptor

## References

[1] Sgouros G., Bodei L., McDevitt M.R., Nedrow J.R. (2020). Radiopharmaceutical therapy in cancer: Clinical advances and challenges. Nat. Rev. Drug Discov..

[2] Stokkel M.P.M., Gotthardt M., Herrmann K., Gnanasegaran G. (2025). Theranostics in Perspective: White Paper. J. Nucl. Med..

[3] Sun J., Huangfu Z., Yang J., Wang G., Hu K., Gao M., Zhong Z. (2022). Imaging-guided targeted radionuclide tumor therapy: From concept to clinical translation. Adv. Drug Deliv. Rev..

[4] Shah A., Dabhade A., Bharadia H., Parekh P.S., Yadav M.R., Chorawala M.R. (2024). Navigating the landscape of theranostics in nuclear medicine: Current practice and future prospects. Z. Naturforsch. C.

[5] Dickson J.C., Armstrong I.S., Minguez Gabina P., Denis-Bacelar A., Krizsan A.K., Gear J.M., Wyngaert T.V.D., de Geus-Oei L.-F., Herrmann K. (2023). EANM practice guideline for quantitative SPECT-CT. Eur. J. Nucl. Med. Mol. Imaging.

[6] Zanzonico P. (2025). Radiation dosimetry in theranostics: A review. J. Nucl. Med. Technol..

[7] Strigari L., Konijnenberg M., Chiesa C., Bardies M., Du Y., Gleisner K.S., Lassmann M., Flux G. (2014). The evidence base for the use of internal dosimetry in clinical molecular radiotherapy. Eur. J. Nucl. Med. Mol. Imaging.

[8] Bodey R.K., Flux G.D., Evans P.M. (2003). Combining dosimetry for targeted radionuclide and external beam therapies using the biologically effective dose. Cancer Biother. Radiopharm..

[9] Bodey R.K., Flux G.D., Evans P.M. (2004). Application of the linear-quadratic model to combined modality radiotherapy. Int. J. Radiat. Oncol. Biol. Phys..

[10] Hobbs R.F., McNutt T., Baechler S., He B., Esaias C.E., Frey E.C., Loeb D.M., Wahl R.L., Shokek O., Sgouros G. (2011). A treatment planning method for sequentially combining radiopharmaceutical therapy and external radiation therapy. Int. J. Radiat. Oncol. Biol. Phys..

[11] Cremonesi M., Ferrari M., Botta F., Guerriero F., Garibaldi C., Bodei L., De Cicco C., Grana C.M., Pedroli G., Orecchia R. (2014). Planning combined treatments of external beam radiation therapy and molecular radiotherapy. Cancer Biother. Radiopharm..

[12] Abbott E., Falzone N., Lenzo N., Vallis K. (2021). Combining external beam radiation and radionuclide therapies: Rationale, radiobiology, results, and roadblocks. Clin. Oncol..

[13] Parker C., Nilsson S., Heinrich D., Helle S.I., O’Sullivan J.M., Fossa S.D., Chodacki A., Wiechno P., Logue J., Seke M. (2013). Alpha emitter radium-223 and survival in metastatic prostate cancer. N. Engl. J. Med..

[14] Feuerecker B., Tauber R., Knorr K., Heck M., Beheshti A., Seidl C., Bruchertseifer F., Pickhard A., Gafita A., Kratochwil C. (2021). Activity and adverse events of actinium-225-PSMA-617 in advanced metastatic castration-resistant prostate cancer after failure of lutetium-177-PSMA. Eur. Urol..

[15] Tagawa S.T., Thomas C., Sartor A.O., Sun M., Stangl-Kremser J., Bissassar M., Vallabhajosula S., Castellanos S.H., Nauseef J.T., Sternberg C.N. (2023). Prostate-specific membrane antigen-targeting alpha emitter via antibody delivery for metastatic castration-resistant prostate cancer: A phase I dose-escalation study of Ac-225-J591. J. Clin. Oncol..

[16] Sathekge M.M., Lawal I.O., Bal C., Bruchertseifer F., Ballal S., Cardaci G., Davis C., Eiber M., Hekimsoy T., Knoesen O. (2024). Actinium-225-PSMA radioligand therapy of metastatic castration-resistant prostate cancer (WARMTH Act): A multicentre, retrospective study. Lancet Oncol..

[17] Fu H., Huang J., Zhao T., Wang H., Chen Y., Xu W., Pang Y., Guo W., Sun L., Wu H. (2023). Fibroblast activation protein-targeted radioligand therapy with Lu-177-EB-FAPI for metastatic radioiodine-refractory thyroid cancer: First-in-human, dose-escalation study. Clin. Cancer Res..

[18] Liu H., Guo R., Zhang X., Ji H., Sun S., Sun S., Liu J., Yang Z., Wang R. (2026). Safety and efficacy of Lu-177-FAPI-XT radioligand therapy in advanced sarcoma and other cancer entities: First-in-human, dose-escalation study. Eur. J. Nucl. Med. Mol. Imaging.

[19] Van Laere C., Koole M., Deroose C.M., Van de Voorde M., Baete K., Cocolios T.E., Duchemin C., Ooms M., Cleeren F. (2024). Terbium radionuclides for theranostic applications in nuclear medicine: From atom to bedside. Theranostics.

[20] Buteau J.P., Kostos L., Jackson P.A., Xie J., Haskali M.B., Alipour R., McIntosh L.E., Emmerson B., MacFarlane L., Martin C.A. (2025). First-in-human results of Tb-161-PSMA-I&T dual beta-Auger radioligand therapy in metastatic castration-resistant prostate cancer (VIOLET): A single-centre, phase 1/2 study. Lancet Oncol..

[21] Strosberg J., El-Haddad G., Wolin E., Hendifar A., Yao J., Chasen B., Mittra E., Kunz P.L., Kulke M.H., Jacene H. (2017). Phase 3 trial of Lu-177-dotatate for midgut neuroendocrine tumors. N. Engl. J. Med..

[22] Strosberg J.R., Caplin M.E., Kunz P.L., Ruszniewski P.B., Bodei L., Hendifar A., Mittra E., Wolin E.M., Yao J.C., Pavel M.E. (2021). Lu-177-dotatate plus long-acting octreotide versus high-dose long-acting octreotide in midgut neuroendocrine tumours (NETTER-1): Final overall survival and long-term safety results. Lancet Oncol..

[23] U.S. Food and Drug Administration (2018). FDA Approves Lutetium Lu 177 Dotatate for Treatment of GEP-NETs.

[24] Sartor O., de Bono J., Chi K.N., Fizazi K., Herrmann K., Rahbar K., Tagawa S.T., Nordquist L.T., Vaishampayan N., El-Haddad G. (2021). Lutetium-177-PSMA-617 for metastatic castration-resistant prostate cancer. N. Engl. J. Med..

[25] Morris M.J., Castellano D., Herrmann K., de Bono J.S., Shore N.D., Chi K.N., Crosby M., Piulats J.M., Fléchon A., Wei X.X. (2024). Lu-177-PSMA-617 versus a change of androgen receptor pathway inhibitor therapy for taxane-naïve patients with progressive metastatic castration-resistant prostate cancer (PSMAfore): A phase 3 randomized trial. Lancet.

[26] U.S. Food and Drug Administration (2025). FDA Expands Pluvicto’s Metastatic Castration-Resistant Prostate Cancer Indication. https://www.fda.gov/drugs/resources-information-approved-drugs/fda-expands-pluvictos-metastatic-castration-resistant-prostate-cancer-indication.

[27] Fizazi K., Morris M.J., Shore N.D., Chi K.N., Crosby M., de Bono J.S., Herrmann K., Roubaud G., Nagarajah J., Fleming M. (2025). Health-related quality of life, pain, and symptomatic skeletal events with Lu-177-PSMA-617 in patients with progressive metastatic castration-resistant prostate cancer (PSMAfore): An open-label, randomized, phase 3 trial. Lancet Oncol..

[28] Giovanella L., Tuncel M., Aghaee A., Campenni A., De Virgilio A., Petranovic Ovcaricek P. (2024). Theranostics of thyroid cancer. Semin. Nucl. Med..

[29] Gulec S.A., Benites C., Cabanillas M.E. (2024). Molecular perspectives in radioactive iodine theranostics: Current redifferentiation protocols for mis-differentiated thyroid cancer. J. Clin. Med..

[30] U.S. Food and Drug Administration (2018). FDA Approves Iobenguane I 131 for Rare Adrenal Gland Tumors.

[31] Pryma D.A., Chin B.B., Noto R.B., Dillon J.S., Perkins S., Solnes L., Kostakoglu L., Serafini A.N., Pampaloni M.H., Jensen J. (2019). Efficacy and safety of high-specific-activity I-131-MIBG therapy in patients with advanced pheochromocytoma or paraganglioma. J. Nucl. Med..

[32] U.S. Food and Drug Administration (2026). FDA Approves Lutetium Lu 177 Vipivotide Tetraxetan with Androgen Receptor Pathway Inhibitor Therapy for Metastatic Androgen Pathway Modulation-Naïve or -Sensitive Prostate Cancer. https://www.fda.gov/drugs/resources-information-approved-drugs/fda-approves-lutetium-lu-177-vipivotide-tetraxetan-androgen-receptor-pathway-inhibitor-therapy.

[33] van der Sar E.C.A., Braat A.J.A.T., van der Voort-van Zyp J.R.N., van der Veen B.S., van Leeuwen P.J., de Vries-Huizing D.M.V., Hendrikx J.M.A., Lam M.G.E.H., Vogel W.V. (2023). Tolerability of concurrent external beam radiotherapy and [177Lu]Lu-PSMA-617 for node-positive prostate cancer in treatment naïve patients, phase I study (PROQURE-I trial). BMC Cancer.

[34] Grkovski M., O’Donoghue J.A., Imber B.S., Andl G., Tu C., Lafontaine D., Schwartz J., Thor M., Zelefsky M.J., Humm J.L. (2023). Lesion dosimetry for Lu-177-PSMA-617 radiopharmaceutical therapy combined with stereotactic body radiotherapy in oligometastatic castration-sensitive prostate cancer. J. Nucl. Med..

[35] Teunissen F.R., Oprea-Lager D.E., Peters S.M.B., Smeenk R.J., Heskamp S., Bussink J. (2025). Current developments in combining external-beam radiotherapy and Lu-177-labeled PSMA ligands for prostate cancer treatment. J. Nucl. Med..

[36] Reissig F., Wunderlich G., Runge R., Freudenberg R., Luhr A., Kotzerke J. (2020). The effect of hypoxia on induction of strand breaks in plasmid DNA by alpha-, beta- and Auger electron-emitters Ra-223, Re-188, Tc-99m and DNA-binding Tc-99m-labeled pyrene. Nucl. Med. Biol..

[37] Murray I., Flux G. (2021). Applying radiobiology to clinical molecular radiotherapy. Nucl. Med. Biol..

[38] Verburg F.A., Nonnekens J., Konijnenberg M.W., de Jong M. (2021). To go where no one has gone before: Necessity of radiobiology studies for exploration beyond the Holy Gray in radionuclide therapy. Eur. J. Nucl. Med. Mol. Imaging.

[39] Verburg F.A., Nonnekens J., Konijnenberg M.W., de Jong M. (2021). The limits of the Holy Gray in radioembolization and beyond. Eur. J. Nucl. Med. Mol. Imaging.

[40] Gholami Y.H., Willowson K.P., Forwood N.J., Harvie R., Hardcastle N., Bromley R., Ryu H., Yuen S., Howell V.M., Kuncic Z. (2018). Comparison of radiobiological parameters for Y-90 radionuclide therapy and external beam radiotherapy in vitro. EJNMMI Phys..

[41] Lee B.Q., Abbott E.M., Able S., Thompson J.M., Hill M.A., Kartsonaki C., Vallis K.A., Falzone N. (2019). Radiosensitivity of colorectal cancer to 90Y and the radiobiological implications for radioembolisation therapy. Phys. Med. Biol..

[42] Heynickx N., Herrmann K., Vermeulen K., Baatout S., Aerts A. (2021). The salivary glands as a dose limiting organ of PSMA-targeted radionuclide therapy: A review. Nucl. Med. Biol..

[43] Kesavan M., Turner J.H. (2016). Myelotoxicity of peptide receptor radionuclide therapy of neuroendocrine tumors: A decade of experience. Cancer Biother. Radiopharm..

[44] Strosberg J., Hofman M.S., Al-Toubah T., Hope T.A. (2024). Rethinking dosimetry: The perils of extrapolated external-beam radiotherapy constraints to radionuclide therapy. J. Nucl. Med..

[45] Tran-Gia J., Cicone F., Koole M., Verburg F.A., Konijnenberg M., Krause B.J., Lassmann M., Stokke C. (2025). Rethinking dosimetry: A European perspective. J. Nucl. Med..

[46] Wang Y., Barmin R., Mottaghy F.M., Kiessling F., Lammers T., Pallares R.M. (2025). Nanoparticles in nuclear medicine: From diagnostics to therapeutics. J. Control. Release.

[47] Moreno-Alcantar G., Drexler M., Casini A. (2024). Assembling a new generation of radiopharmaceuticals with supramolecular theranostics. Nat. Rev. Chem..

[48] Vergnaud L., Dewaraja Y.K., Giraudet A.L., Badel J.N., Sarrut D. (2024). A review of Lu-177 dosimetry workflows: How to reduce the imaging workloads?. EJNMMI Phys..

[49] Chen X., Lu J., Jiang X., Afshar-Oromieh A., Rominger A., Shi K., Mok G.S.P. (2023). Lu-177-PSMA dosimetry for kidneys and tumors based on SPECT images at two imaging time points. Front. Oncol..

[50] Bolch W.E., Eckerman K.F., Sgouros G., Thomas S.R. (2009). MIRD pamphlet No. 21: A generalized schema for radiopharmaceutical dosimetry—Standardization of nomenclature. J. Nucl. Med..

[51] Ljungberg M., Celler A., Konijnenberg M.W., Eckerman K.F., Dewaraja Y.K., Sjögreen-Gleisner K. (2016). MIRD pamphlet No. 26: Joint EANM/MIRD guidelines for quantitative Lu-177 SPECT applied for dosimetry of radiopharmaceutical therapy. J. Nucl. Med..

[52] Sjögreen Gleisner K., Chouin N., Mínguez Gabiña P., Cicone F., Gnesin S., Stokke C., Konijnenberg M., Cremonesi M., Verburg F.A., Bernhardt P. (2022). EANM dosimetry committee recommendations for dosimetry of Lu-177-labelled somatostatin-receptor- and PSMA-targeting ligands. Eur. J. Nucl. Med. Mol. Imaging.

[53] Gear J.I., Cox M.G., Gustafsson J., Sjögreen Gleisner K., Murray I., Glatting G., Konijnenberg M., Flux G.D. (2018). EANM practical guidance on uncertainty analysis for molecular radiotherapy absorbed dose calculations. Eur. J. Nucl. Med. Mol. Imaging.

[54] Currie G.M., Rohren E. (2025). The role of artificial intelligence in theranostics. J. Nucl. Med. Technol..

[55] Papachristou K., Panagiotidis E., Makridou A., Kalathas T., Masganis V., Paschali A., Aliberti M., Chatzipavlidou V. (2023). Artificial intelligence in nuclear medicine physics and imaging. Hell. J. Nucl. Med..

[56] Kawamura M., Kamomae T., Yanagawa M., Kamagata K., Fujita S., Ueda D., Matsui Y., Fushimi Y., Fujioka T., Nozaki T. (2024). Revolutionizing radiation therapy: The role of artificial intelligence in clinical practice. J. Radiat. Res..

[57] Brock K.K., Mutic S., McNutt T.R., Li H., Kessler M.L. (2017). Use of image registration and fusion algorithms and techniques in radiotherapy: Report of the AAPM Radiation Therapy Committee Task Group No. 132. Med. Phys..

[58] Wang T.H., Huang P.I., Hu Y.W., Lin K.H., Liu C.S., Lin Y.Y., Liu C.A., Tseng H.S., Liu Y.M., Lee R.C. (2018). Combined Yttrium-90 microsphere selective internal radiation therapy and external beam radiotherapy in hepatocellular carcinoma: From clinical aspects to dosimetry. PLoS ONE.

[59] Abbott E., Young R.S., Hale C., Mitchell K., Falzone N., Vallis K.A., Kennedy A. (2021). Stereotactic inverse dose planning after yttrium-90 selective internal radiation therapy in hepatocellular cancer. Adv. Radiat. Oncol..

[60] Van B.J., Dewaraja Y.K., Sangogo M.L., Mikell J.K. (2021). Y-90 selective internal radiation therapy: Evaluation of tumor control probability variation across dosimetric models. EJNMMI Phys..

[61] Adam D.P., Grudzinski J.J., Marsh I.R., Hill P.M., Cho S.Y., Bradshaw T.J., Longcor J., Burr A., Bruce J.Y., Harari P.M. (2024). Voxel-level dosimetry for combined iodine-131 radiopharmaceutical therapy and external beam radiotherapy treatment paradigms of head and neck cancer. Int. J. Radiat. Oncol. Biol. Phys..

[62] Bruce J.Y., Burr A., Kimple R.J., Adam D.P., Yu M., Piaskowski S.M., Glazer T.A., Hill P., Hartig G.K., McCulloch T.M. (2025). Safety and toxicity of iopofosine I-131 (CLR 131) with external beam radiation therapy in recurrent or metastatic head and neck cancer: Results of a phase 1 single-centre open-label study. eBioMedicine.

[63] Kreissl M.C., Hanscheid H., Lohr M., Verburg F.A., Schiller M., Lassmann M., Reiners C., Samnick S.S., Buck A.K., Flentje M. (2012). Combination of peptide receptor radionuclide therapy with fractionated external beam radiotherapy for treatment of advanced symptomatic meningioma. Radiat. Oncol..

[64] Hartrampf P.E., Hanscheid H., Kertels O., Schirbel A., Kreissl M.C., Flentje M., Sweeney R.A., Buck A.K., Polat B., Lapa C. (2020). Long-term results of multimodal peptide receptor radionuclide therapy and fractionated external beam radiotherapy for treatment of advanced symptomatic meningioma. Clin. Transl. Radiat. Oncol..

[65] Burdick M.J., Neumann D., Pohlman B., Reddy C.A., Tendulkar R.D., Macklis R. (2011). External beam radiotherapy followed by Y-90 ibritumomab tiuxetan in relapsed or refractory bulky follicular lymphoma. Int. J. Radiat. Oncol. Biol. Phys..

[66] Baczyk M., Milecki P., Pisarek M., Gut P., Antczak A., Hrab M. (2013). A prospective randomized trial: A comparison of the analgesic effect and toxicity of Sm-153 radioisotope treatment in monotherapy and combined therapy including local external beam radiotherapy among metastatic castration-resistant prostate cancer patients with painful bone metastases. Neoplasma.

[67] Hasan H., Deek M.P., Phillips R., Hobbs R.F., Malek R., Radwan N., Kiess A.P., Dipasquale S., Huang J., Caldwell T. (2020). A phase II randomized trial of radium-223 dichloride and SABR versus SABR for oligometastatic prostate cancer (RAVENS). BMC Cancer.

[68] Wang J.H., Sherry A.D., Bazyar S., Sutera P., Radwan N., Phillips R.M., Deek M.P., Lu J., Dipasquale S., Deville C. (2025). Outcomes of radium-223 and stereotactic ablative radiotherapy versus stereotactic ablative radiotherapy for oligometastatic prostate cancers: The RAVENS phase II randomized trial. J. Clin. Oncol..

[69] Anderson P.M., Scott J., Parsai S., Zahler S., Worley S., Shrikanthan S., Subbiah V., Murphy E. (2020). Ra-223 for metastatic osteosarcoma: Combination therapy with other agents and external beam radiotherapy. ESMO Open.

[70] International Commission on Radiation Units and Measurements (2010). Prescribing, Recording, and Reporting Photon-Beam Intensity-Modulated Radiation Therapy (IMRT). ICRU Report 83. J. ICRU.

[71] International Commission on Radiation Units and Measurements (2014). Prescribing, Recording, and Reporting of Stereotactic Treatments with Small Photon Beams. ICRU Report 91. J. ICRU.

